# Author Correction: Rice auxin influx carrier *OsAUX1* facilitates root hair elongation in response to low external phosphate

**DOI:** 10.1038/s41467-018-04280-y

**Published:** 2018-05-01

**Authors:** Jitender Giri, Rahul Bhosale, Guoqiang Huang, Bipin K. Pandey, Helen Parker, Susan Zappala, Jing Yang, Anne Dievart, Charlotte Bureau, Karin Ljung, Adam Price, Terry Rose, Antoine Larrieu, Stefan Mairhofer, Craig J. Sturrock, Philip White, Lionel Dupuy, Malcolm Hawkesford, Christophe Perin, Wanqi Liang, Benjamin Peret, Charlie T. Hodgman, Jonathan Lynch, Matthias Wissuwa, Dabing Zhang, Tony Pridmore, Sacha J. Mooney, Emmanuel Guiderdoni, Ranjan Swarup, Malcolm J. Bennett

**Affiliations:** 1 Centre for Plant Integrative Biology (CPIB), School of Biosciences, University of Nottingham, Nottingham, LE12 3RD UK; 2 National Institute of Plant Genome Research (NIPGR), New Delhi, India; 3 State Key Laboratory of Hybrid Rice, Shanghai Jiao Tong University, Shanghai, China; 4 CIRAD, UMR AGAP, F34398 Montpellier, Cedex 5 France; 5 Umeå Plant Science Centre, Department of Forest Genetics and Plant Physiology, Swedish University of Agricultural Sciences, SE-901 83 Umeå, Sweden; 6 Institute of Biological and Environmental Sciences, University of Aberdeen, AB24 2TZ Aberdeen, UK; 7 Japan International Center for Agricultural Sciences (JIRCAS), 1-1 Ohwashi, Tsukuba, Ibaraki 305-8686 Japan; 8 School of Computer Science, University of Nottingham, Jubilee Campus, Nottingham, NG8 1BB UK; 9 Ecological Sciences, The James Hutton Institute, Invergowrie, Dundee DD2 5DA UK; 10 Rothamsted Research, Harpenden, Hertfordshire, AL5 2JQ UK; 11 Department of Plant Science, The Pennsylvania State University, 102 Tyson Building, University Park, PA 16802 USA; 12 University of Adelaide-SJTU Joint Centre for Agriculture and Health, University of Adelaide, Waite Campus, Urrbrae, SA Australia; 13 Present Address: Southern Cross Plant Science, Southern Cross University, Lismore, NSW 2480 Australia

Correction to: *Nature Communications* 10.1038/s41467-018-03850-4, published online 12 April 2018

The original version of this Article omitted the following from the Acknowledgements:

‘We also thank DBT-CREST BT/HRD/03/01/2002.’

This has been corrected in both the PDF and HTML versions of the Article.

