# Supplementary material for: Rice auxin influx carrier OsAUX1 facilitates root hair elongation in response to low external phosphate
Source: Nat Commun. 2018 Apr 12;9:1408. doi: 10.1038/s41467-018-03850-4 (PMC5897452; doi:10.1038/s41467-018-03850-4)
Supplement: Supplementary file 1 — Supplementary Information [file 41467_2018_3850_MOESM1_ESM.pdf]

Supplementary Information

Rice auxin influx carrier *OsAUX1* facilitates root hair elongation in response to low external phosphate

Giri et al.

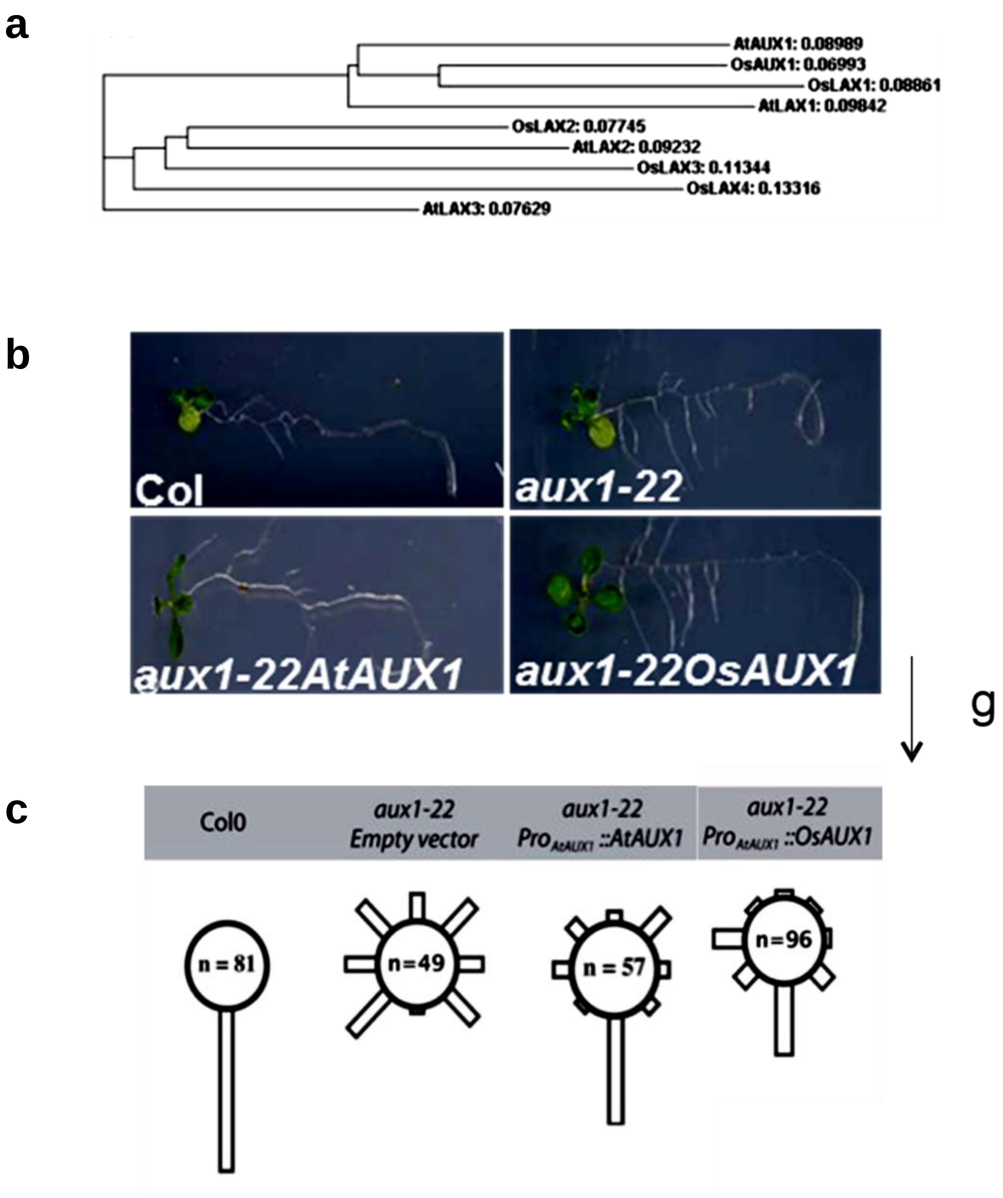

Supplementary Figure 1. Identification and characterization of rice *OsAux1*. **(a)** Phylogenomic tree of the *AUX/LAX* gene family in *Arabidopsis* and rice. (**b)** Functional complementation of the *Arabidopsis aux1-22* mutant line by Arabidopsis *AtAUX1* and rice *OsAUX1* cDNA sequences driven by the AtAUX1 promoter. The seedlings were allowed to grow for three days and then the plates were turned 90° for 24 h. **(c)** Quantification of the direction of root growth of the denoted lines

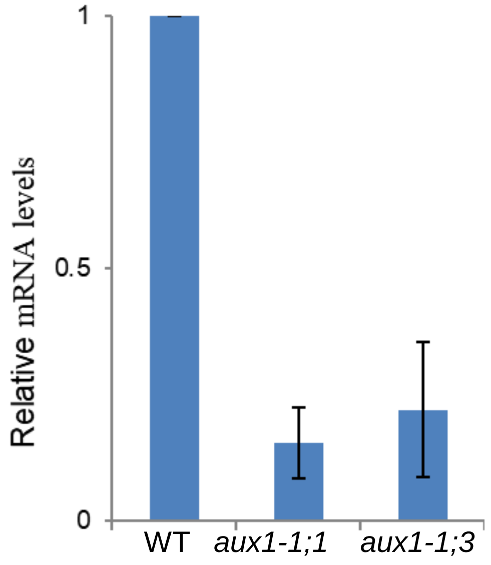

Supplementary Figure 2. T-DNA mutant lines exhibit reduced OsAUX1 levels.
RT- qPCR profiling of Os*AUX1* transcripts in WT, *osaux1-1;1* and *osaux1-1;3* lines revealed a significant reduction in Os*AUX1* mRNA abundance in both mutant alleles. Error bars mean ± SE, n = three biological replicate and four technical replicates of each lines.

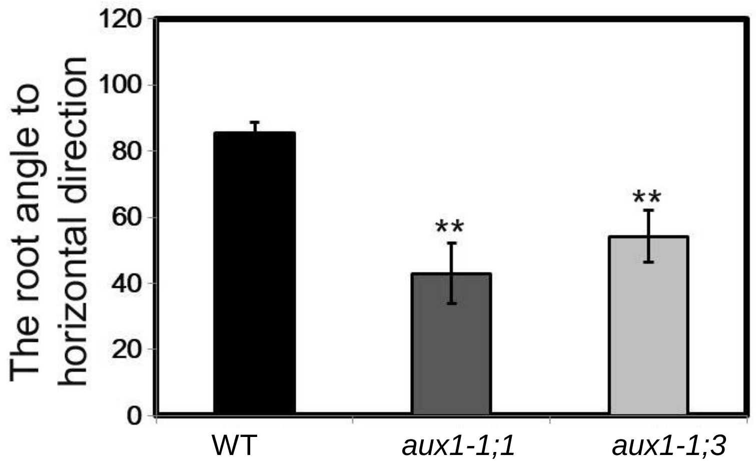

Supplementary Figure 3. Root angle measurement of WT and aux1 seedlings

The graphical representation of root angles of WT and *aux-1* alleles. The root angles were calculated using horizontal line coming from the root emergence point as 0 degree at the initiation site for WT, *aux1-1;1* and *aux1-1;3* after 6-day's growth on plates. All crown and primary roots were included for angle measurement. Error bars represents means ± SD, n = 11, two asterisks mean significant differences (p < 0.01 from Student's t-test).

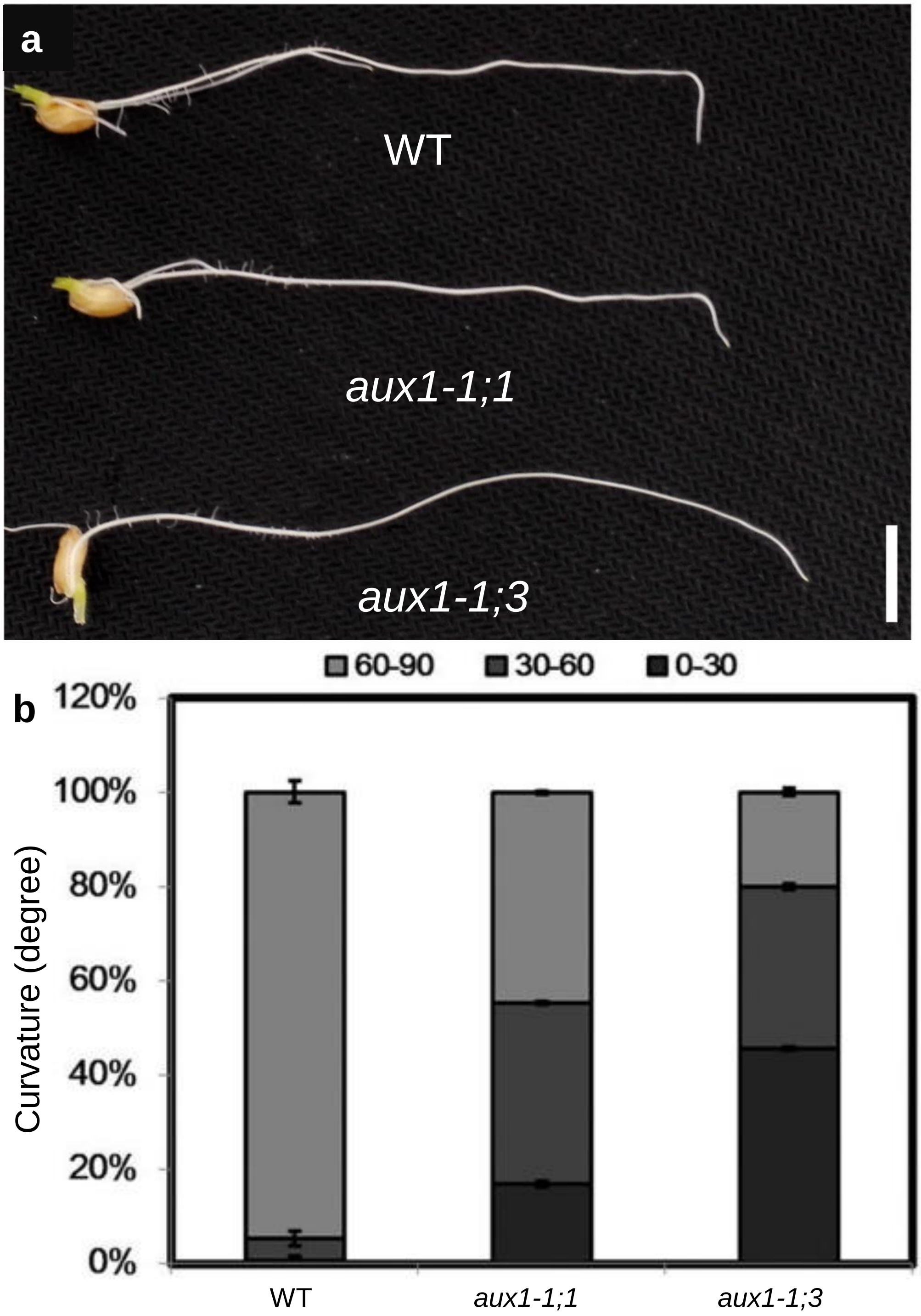

Supplementary Figure 4. *OsAUX1* mutants exhibit defective root gravitropic responses.

(a) Representative images of WT, *aux1-1;1* and *aux1-1;3* after 8-h gravity stimulation. Scale bar, 1 cm. **(b)** The quantified data for the curvature degree. Error bars mean ± SE, n = three independent biological repeats with at least 40 roots analyzed in each assay.

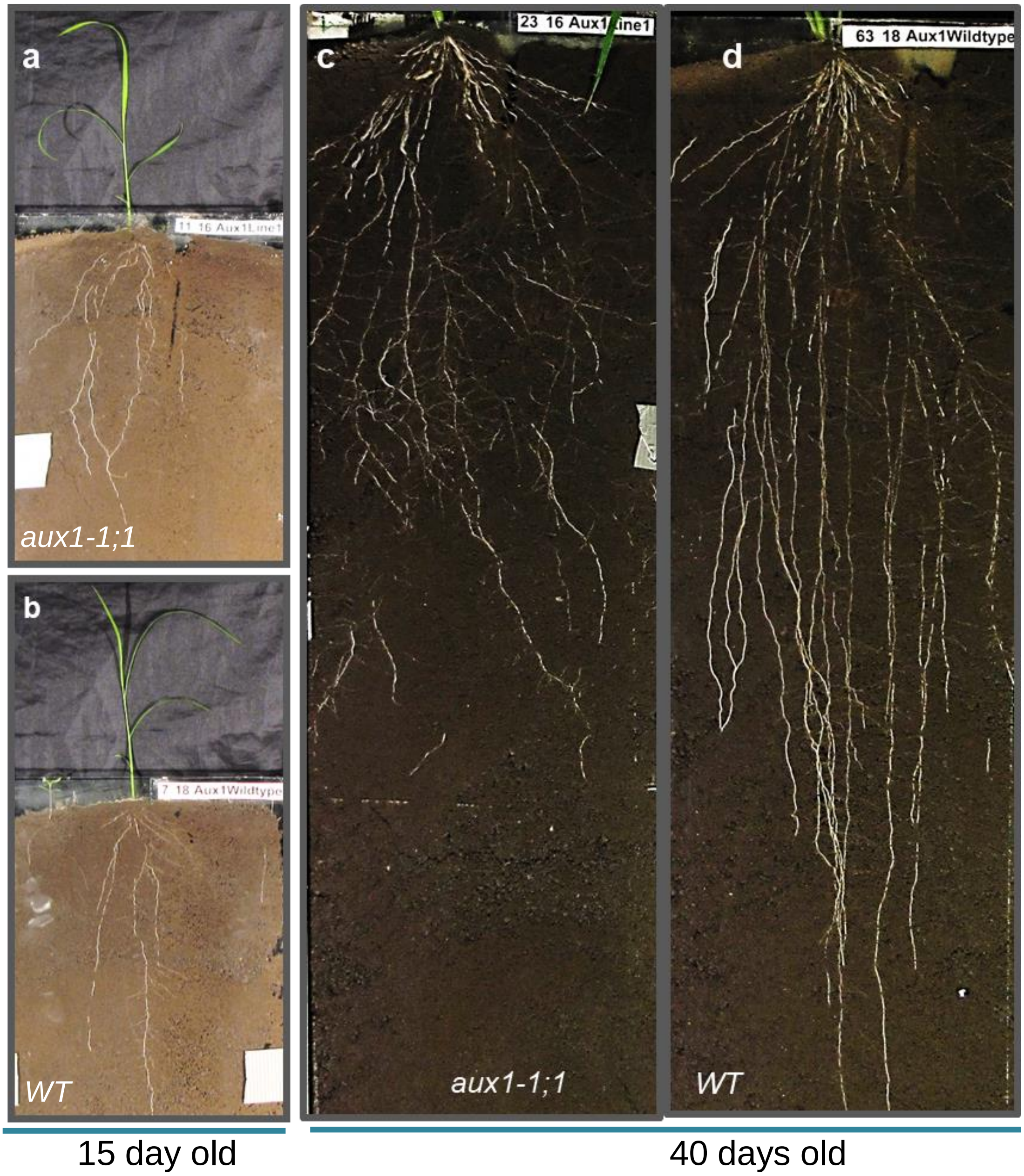

Supplementary Figure 5 Mature *osaux1-1;1* rice plants exhibit reduced root angle. Rice plants were grown in large soil-filled rhizotrons (1.2 x 0.3 x 0.015 m) and representative photographs (four replicates) of the rhizotrons containing 15d (a,b) and 40 d (c,d) old rice plants were taken. The images show that rice *osaux1-1;1* mutants (left a and c) exhibit reduced root angle compared to wild-type (lower left b and extreme right d).

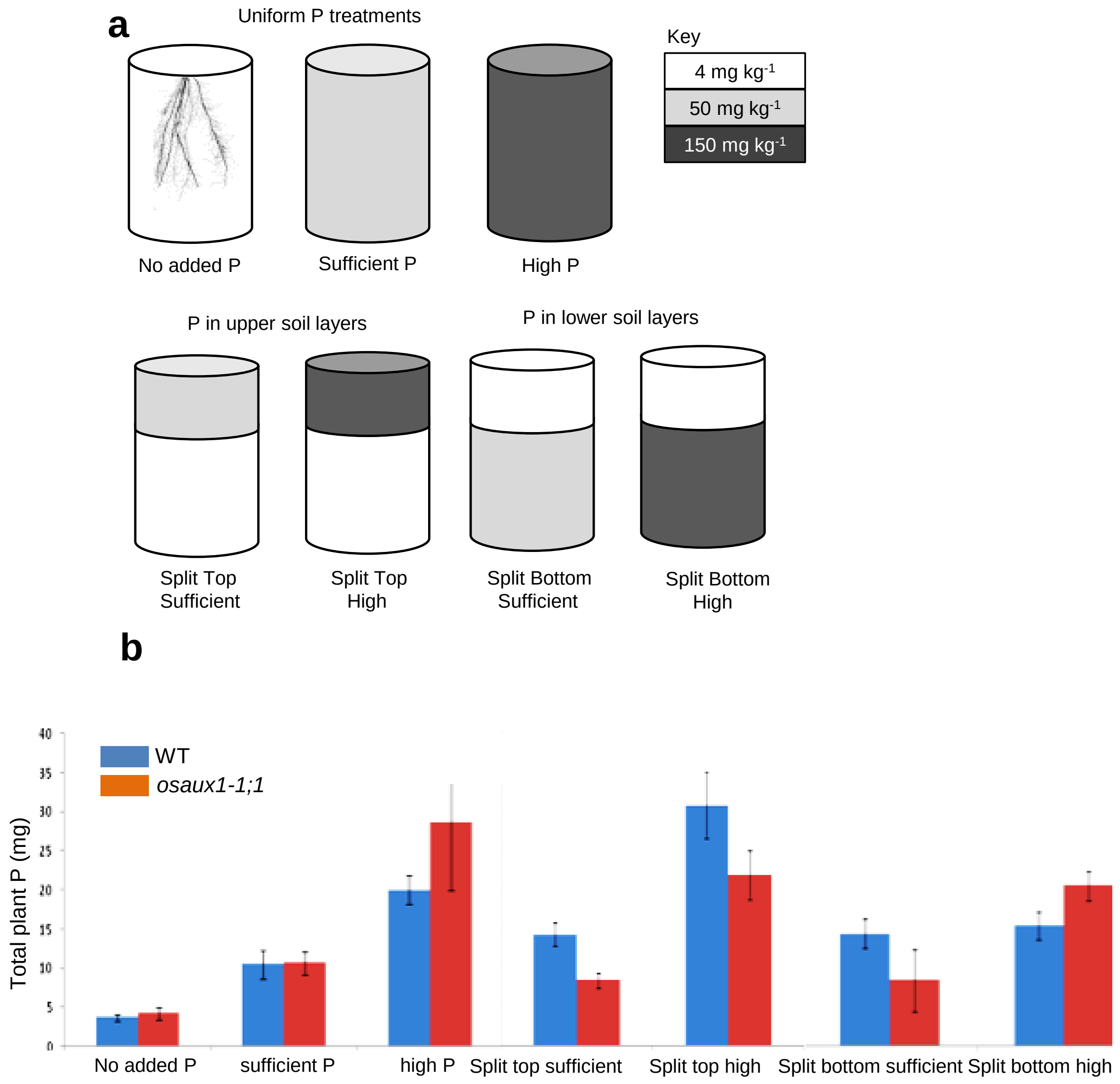

Supplementary Figure 6. Assessing the impact of *OsAUX1* on P foraging in soil.
(a) Experiments used P at three levels (low/no added P, sufficient P and high P) distributed uniformly throughout the soil column (upper panel), in the top layer of soil (bottom left) or in the bottom layers of soil (bottom right). **(b)** Total plant P status in WT and *osaux1-1;1* mutant grown under the different split soil P conditions. Error bars represent standard error (n = 5).

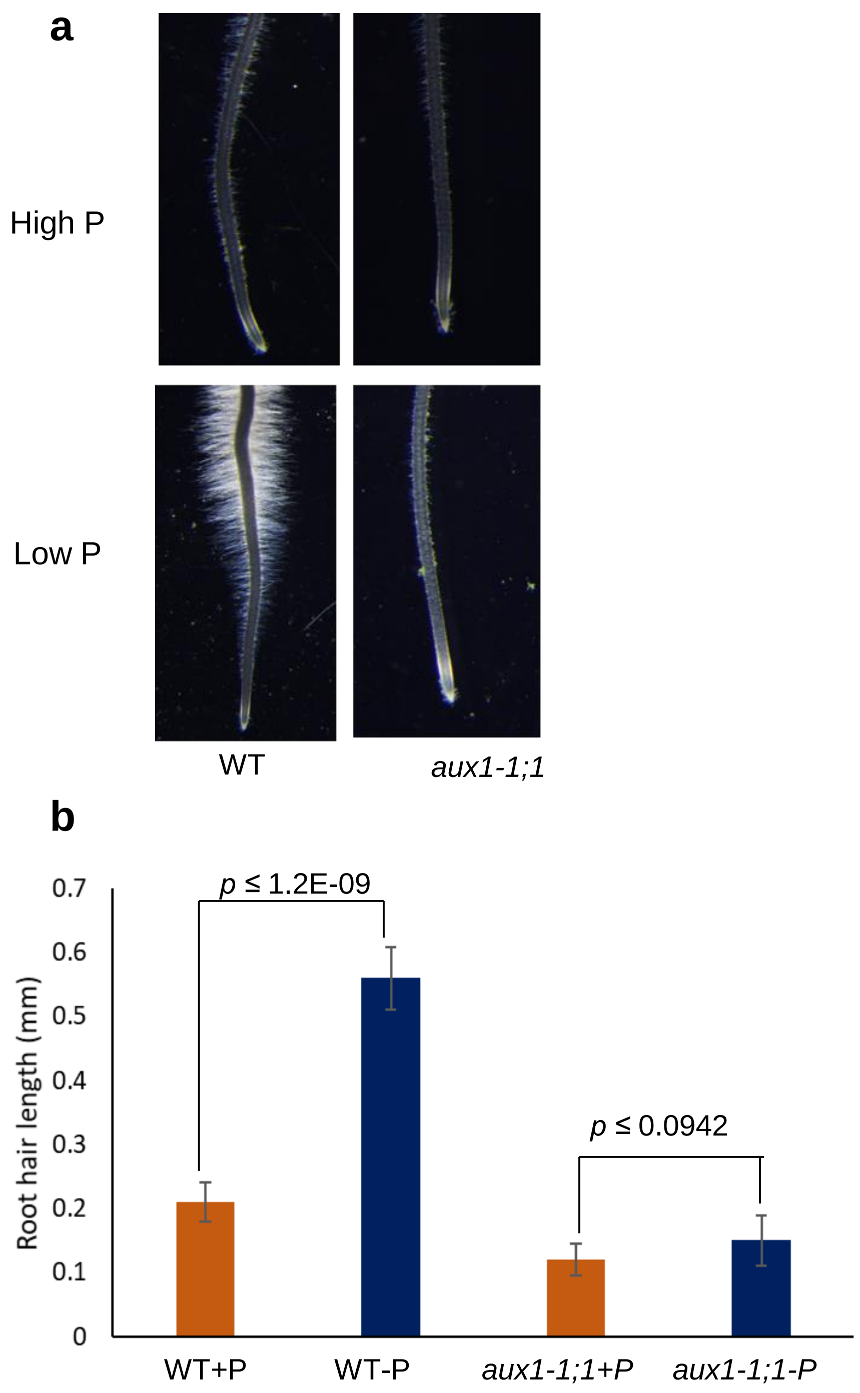

Supplementary Figure 7. Low P root hair growth response is *OsAUX1* dependent
(a) Representative images of WT and *aux1-1;1* root hairs under low and high P conditions
(b) Quantitation of WT and *aux-1-1;1* root hair length under high and low P conditions. Each bar represents at least 10 replicates and each root was analyzed for at least 30 to 50 root hairs on 15 day old seedlings grown for 6 days in hydroponics with three different P levels. *p* value was calculated from Student's *t*-test

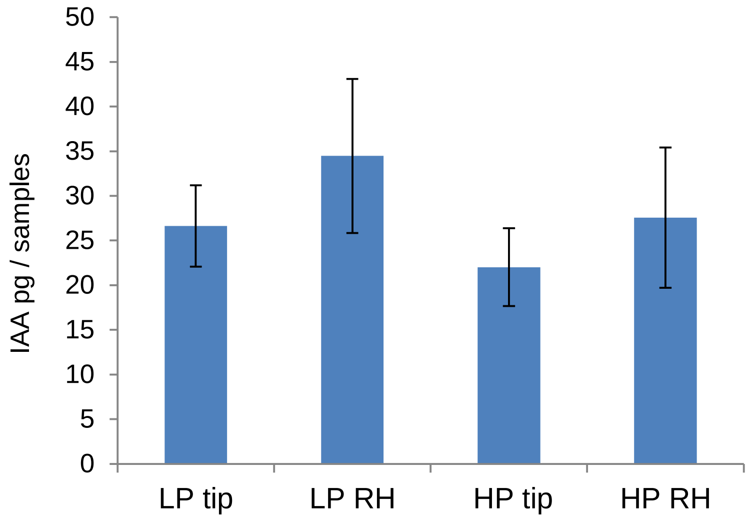

Supplementary Figure 8. IAA quantification in rice root tips and hair zones. Root tip (~1.5 mm) and root hair zone (next 2 mm region) from 15-days-old rice seedlings either grown under low P (3 µM) and high P (312 µM) conditions for 6 days, then were excised under a dissecting stereo microscope, frozen, then analysed using LC-MS/MS. Error bars represents mean ± SE, n = four biological replicates with at least 12-15 roots for each sample.

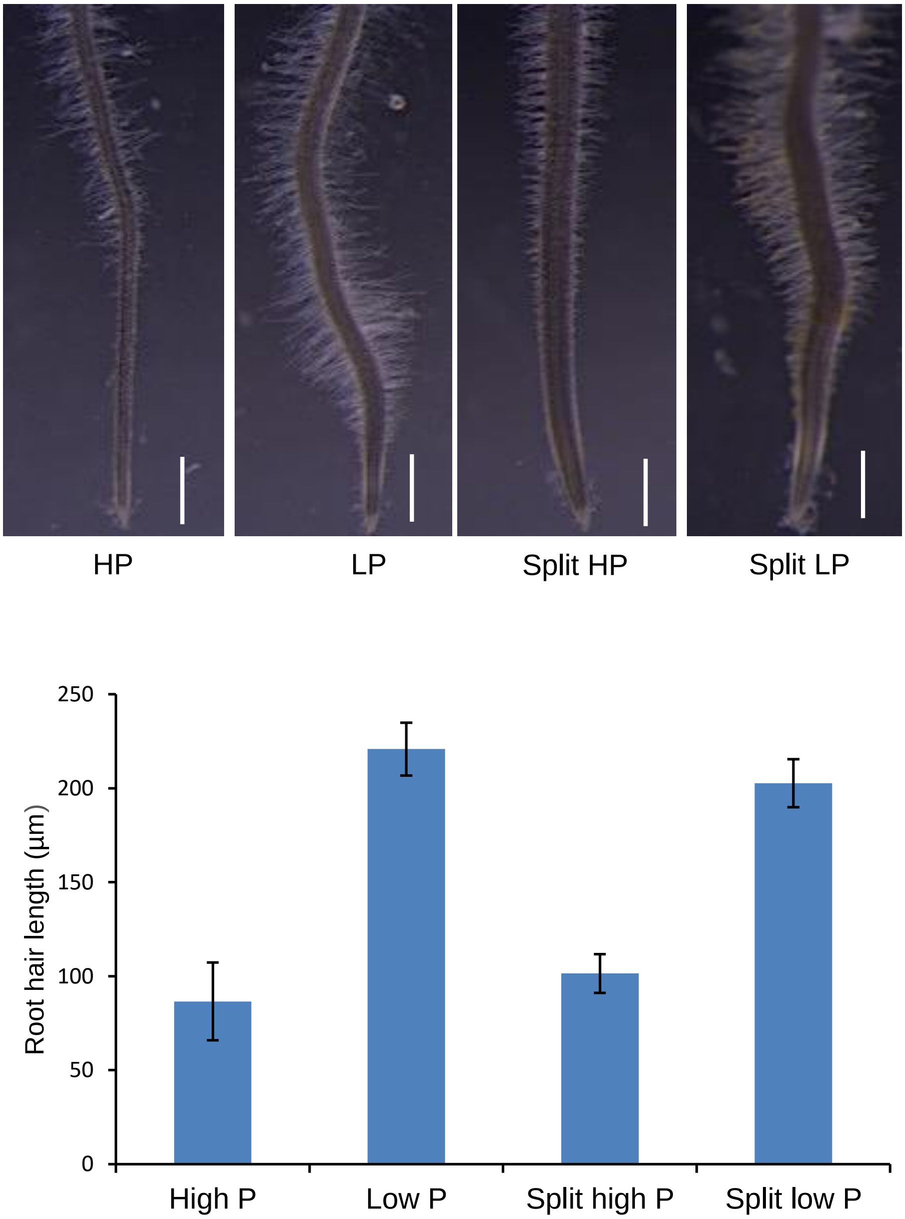

Supplementary Figure 9. Root hair growth is regulated by local P availability. Root hair length under high, low P and split P conditions shown by representative images (top line) and quantitation (lower line). Seedlings (4 days old) were transferred to low and high P Yoshida nutrient media. After 6 days treatment half of the roots were either placed in high or low P levels for another four days. At least 10 roots were used for each treatment. Scale bar represents 200 µm. Error bars mean ± SE, n = two independent biological repeats with 10 roots analyzed in each assay.

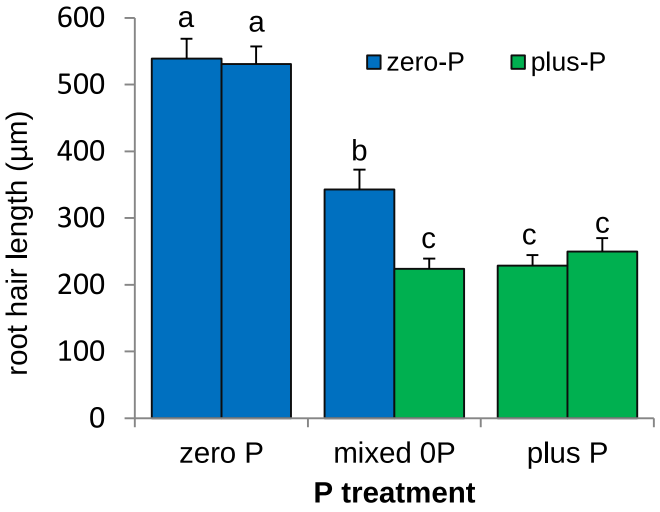

Supplementary Figure 10. Local P levels control root hair length.
Root hair measurement under high, low and mixed P nutrient regimes revealed the importance of local P levels on the regulation of root hair length. The data shown are mean ± SEM. Different letters indicate significant differences ranked by Fisher's Least Significant Difference (LSD) test ($p < 0.05$).

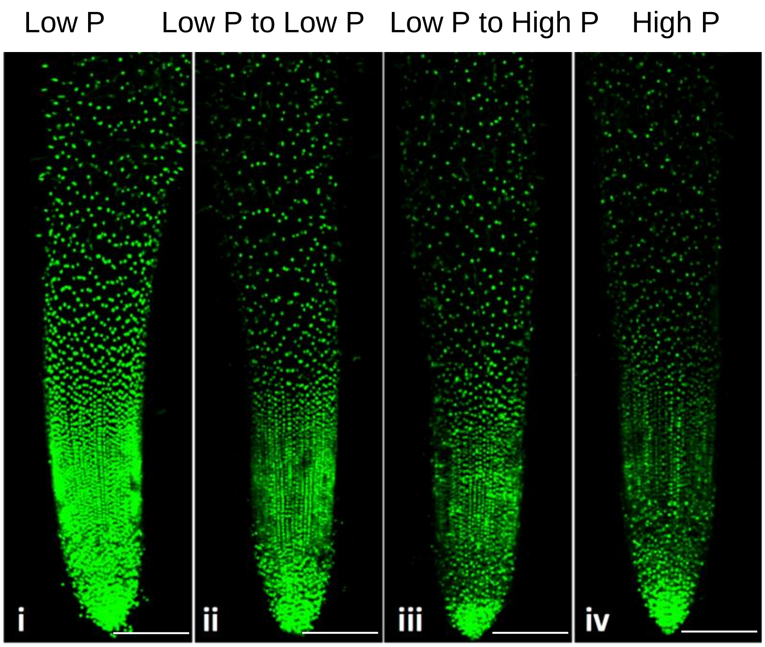

Supplementary Figure 11. Low P induced root auxin is independent of plant P status.

Maximum projection confocal images of Z stacks of *DR5::VENUS* fluorescence in seedlings initially grown at low P for 7 days and then transferred to low P medium (i) for a further 6 days. (ii) and (iii) show *DR5::VENUS* fluorescence for split P roots where 7 day old low P roots were divided into two halves: one half was grown in low (ii) and the other in high P media (iii) for a further 6 days**.** (iv) Maximum projection confocal image of 13 days old high P grown rice roots. Scale bar represents 100 µm.

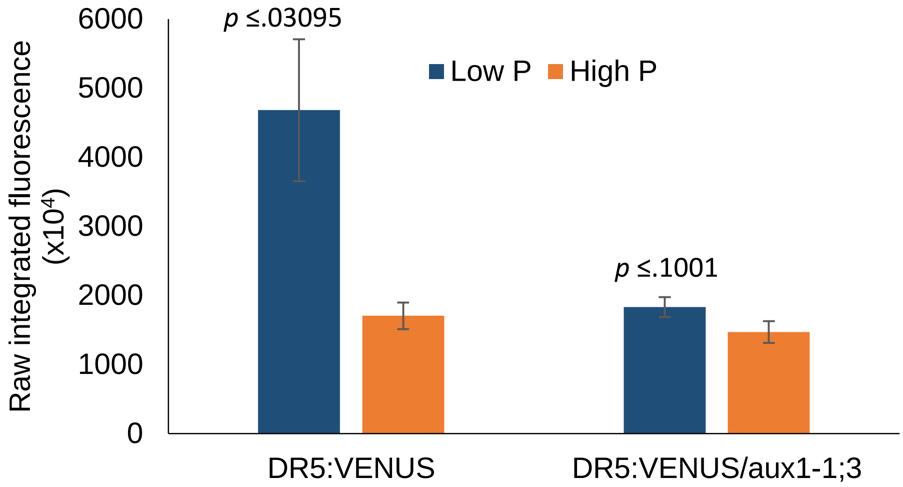

Supplementary Figure 12. *Osaux1-1;3* disrupts the low P induced auxin response.

Raw integrated fluorescence intensity of DR5:VENUS and DR5:VENUS/*aux1-1;3* under low and high P conditions were analyzed through Fiji. Each bar represents the average fluorescence intensity of at least 10 roots under low P and high P grown DR5-VENUS and DR5:VENUS/*aux1-1;3* . *p* values are represented over the top of graph bar showing significance test. Respective *p* values and Student's *t*-test was performed between low and high *p* values of fluorescence intensities.

Supplementary Table 1 Primer sequences used for cDNA amplification of rice *AUX1* and *LAX* genes, *AUX1* promoter amplification and *AUX1* qPCR studies

Primer name		Sequence
**Full length cDNA amplification**		
OsAUX1	Forward	atctcgaggtgccgcgcgagcaggcggag
	Reverse	atggatccgtggtgcggcaatggcaccggcg
OsLAX1	Forward	atctcgaggtgccggccggcgaccaggc
	Reverse	atggatccgtggcgcggcggagccggca
OsLAX2	Forward	tactcgagggctcggcggctgacgggagc
	Reverse	atggatccgagcccgtggctgtgatgccgg
OsLAX3	Forward	gcgtccgggagcagcggcgg
	Reverse	atggatccgaggccgtggcggtggcggtggtgccg
OsLAX4	Forward	atctcgaggcgtcggagaaggtggagacga
	Reverse	atagatctgtgtcttggaggcactggtag
**Promoter region amplification**		
OsAUX1	Forward	taggatcctagctgctttcgggagaga
	Reverse	tactcgaggtcgtcagctgcgtgagt
**Primer Sequences for qRT-PCR**		
OsAUX1	Forward	gcccacatcctcacctacc
	Reverse	gatgaacatgttgagcacgaa
OsActin	Forward	cgaaacgctcagcaccaatg
	Reverse	actcctcgtctcgaccttgc
